# Multiscale performance analysis and optimization of a composite clamp plate for leaf spring assembly considering fiber orientation distribution

**DOI:** 10.1038/s41598-025-13345-0

**Published:** 2025-08-02

**Authors:** Feng Wang, Wenbo Luo, Bo Zou, Jun Yang, Anmin Huang

**Affiliations:** 1 College of Mechanical Engineering and Mechanics, Xiangtan University, Xiangtan, 411105 China; 2 Zhuzhou Time New Material Technology Co., Ltd, Zhuzhou, 412007 China; 3 School of Civil Engineering, Changsha University, Changsha, 410022 China

**Keywords:** Fiber-reinforced material, Leaf spring clamp plate, Fiber orientation, Injection molding simulation, Multiscale analysis, Constitutive model, Structural materials, Mechanical engineering

## Abstract

The growing demand for lightweight solutions in automotive engineering has propelled the adoption of fiber-reinforced thermoplastic composites, necessitating precise characterization of their process-induced mechanical properties. This study develops an integrated multiscale methodology addressing injection-molding-induced fiber orientation heterogeneity in structural components. Through synergistic integration of injection molding simulation, mesoscopic constitutive modeling, and macroscopic structural analysis, we systematically investigated failure mechanisms in automotive leaf spring clamp plates. The proposed framework successfully identifies gravitational segregation during vertical molding as the root cause of terminal fracture under operational loads. Subsequent design optimization implements (1) reorientation of the injection direction to horizontal and (2) localized wall thickness reduction from 37 mm to 19.86 mm. These interventions collectively reduce the maximum principal stress by 19% (from 231 MPa to 187 MPa) while achieving a 12.8% mass reduction (from 780 g to 680 g), demonstrating the concurrent enhancement of structural reliability and lightweighting efficacy.

## Introduction

Owing to rapid global urbanization and strict carbon emission objectives, there is a growing demand for lightweight products. Thermoplastic composites are formed by integrating thermoplastic polymers as the matrix phase with high-performance reinforcing materials, and are characterized by high toughness, chemical stability, ease of processing, and other distinctive features. They have the capacity to soften upon heating and re-solidify when cooled, facilitating the efficient production of diverse complex components, enabling remolding and recycling, and presenting extensive application potential in the aviation, automotive, sports equipment, and construction sectors. Currently, the advancement of thermoplastic composites has emerged as a key area of research^1–3^. Specifically, the automotive industry faces mounting pressure to reduce vehicle weight while maintaining structural integrity, particularly for suspension components such as leaf spring clamp plates. Traditional cast iron designs are being replaced by fiber-reinforced plastics (FRP), yet premature failures in field applications reveal critical knowledge gaps in processing-structure-property relationships.

Many studies address the effects of fibers on the mechanical properties of FRPs. Mortazavian et al.^4^ examined the impacts of fiber orientation and anisotropy on the tensile strength and elastic modulus of short fiber reinforced polymer (SFRP) composites, and reported that tensile properties decrease nonlinearly with increasing fiber orientation angle and are affected by shell-core morphology. Dean et al.^5^ employed microcomputed tomography (µCT) to assess the anisotropy of the SFRP sheets and conducted destructive tests to examine the mechanical behavior of the sheets under various loading conditions, and a user-defined macromechanical constitutive model was introduced for the anisotropic, pressure- and temperature-dependent behavior of the short fiber composites. Chebbi et al.^6^ established an anisotropic hyperelastic constitutive model for short glass fiber-reinforced polyamide-6.6, incorporating isotropic matrix behavior and fiber-induced anisotropy, which was confirmed using experimental tensile and bending assessments. Kim et al.^7^ measured the distribution of fiber orientation using image processing techniques for injection-molded products with varying fiber contents. Mouhmid et al.^8^ examined the mechanical behavior of short glass fiber-reinforced polyamide 6,6; assessed the influences of the fiber content, strain rate, and temperature on tensile characteristics; and utilized acoustic emission and SEM to understand damage mechanisms.

Despite the great progress in the mechanical analysis and engineering applications of FRPs, owing to the uncertainty of the microscopic structures of composite materials, numerous challenges associated with design and manufacturing have emerged during the application process. The primary issue is that the fibers present varying orientations at different locations within the product, leading to anisotropy and spatial inhomogeneity, which results in inconsistent mechanical properties throughout various sections of the product. Since there is a great difference between composite materials and homogeneous materials, the constitutive models for composites are considerably more complex than those for homogeneous materials. Therefore, previous theories applicable to homogeneous materials are rendered irrelevant; it is essential to establish multiscale models that align with composite materials.

Two primary methods are currently employed to study the mechanical properties of composite materials: macroscopic mechanical methods and mesoscopic mechanical methods^9–12^. The macroscopic mechanical method is based on a phenomenological point of view, treating the composite material as a macroscopically homogeneous medium, disregarding the interactions among the constituent phases, and focusing solely on the average performance of the composite material. The stress and strain in this approach do not represent the actual stresses and strains of the matrix and the reinforcement phases, but rather average values at the macroscopic scale. On the other hand, the mesomechanics of composite materials establish a quantitative correlation between the macroscopic properties of composites and the characteristics of various phases and their mesostructure, integrating the morphological attributes of microstructures with the macroscopic mechanical properties to bridge two distinct scales. Mesomechanics is an important subdiscipline bridging macroscopic mechanics and micromechanics; it possesses substantial theoretical value and promising engineering applications for investigating mechanics problems that involve cross-scale effects. It represents one of the leading edges in current mechanics research.

Owing to the complexity of the microstructure mechanism of composite materials, the research cycle for only experimentally assessing material properties is protracted. In addition, this approach consumes substantial human and material resources, making it increasingly challenging to achieve a viable solution. Currently, the advancement of computer technology has rendered numerical simulation analysis increasingly significant in research^13^. The mechanical properties of fiber-reinforced composites are associated with various discipline concerns, including machinery, composite materials, flow heat transfer, mechanics and other related expertise. To obtain precise mechanical properties via simulation, reliance on a single software is generally inadequate; it is essential to integrate multiple software programs across various scales to construct models^14–17^.

In this work, the multiscale coupling simulation method was performed via MoldFlow-Digimat-Abaqus. The molding process was simulated using MoldFlow to obtain the fiber orientation distributions. The meso-constitutive model of the material was constructed through Digimat and subsequently validated by an experimental curve. The macroscopic strength analysis of the components was carried out via Abaqus. Taking an automotive leaf spring clamp plate as a case study, a simulation model was developed to investigate the impact of the glass fiber orientation on the mechanical properties of the product, spanning from the material level to the sample level. The failure of the original structure of the leaf spring clamp plate was analyzed, and the product performance was greatly improved through structural optimization and injection molding modifications. The close agreement between the simulation and experimental results demonstrates the predictive capability of this multiscale approach for fiber-reinforced composite structures.

## Multiscale analysis accounting for the impact of the molding process

### Description of the leaf spring clamp plate

**Fig. 1:**
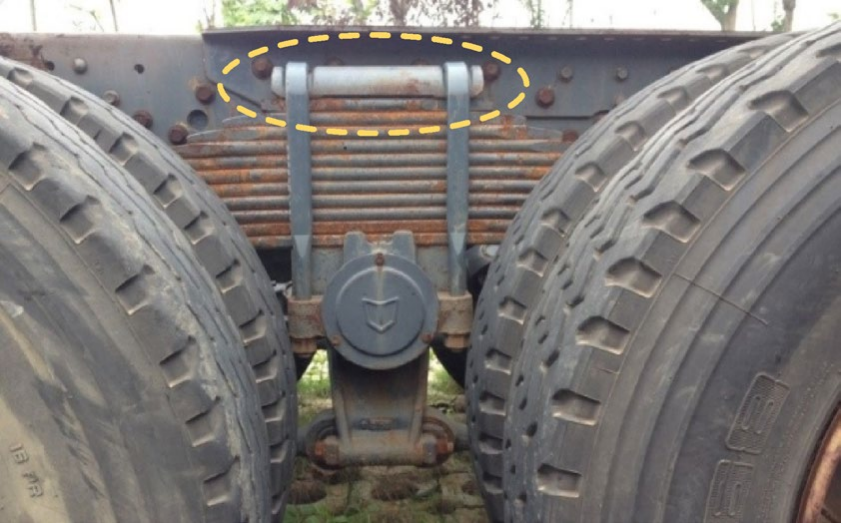
Photo of the metal leaf spring clamp plate assembly.

**Fig. 2:**
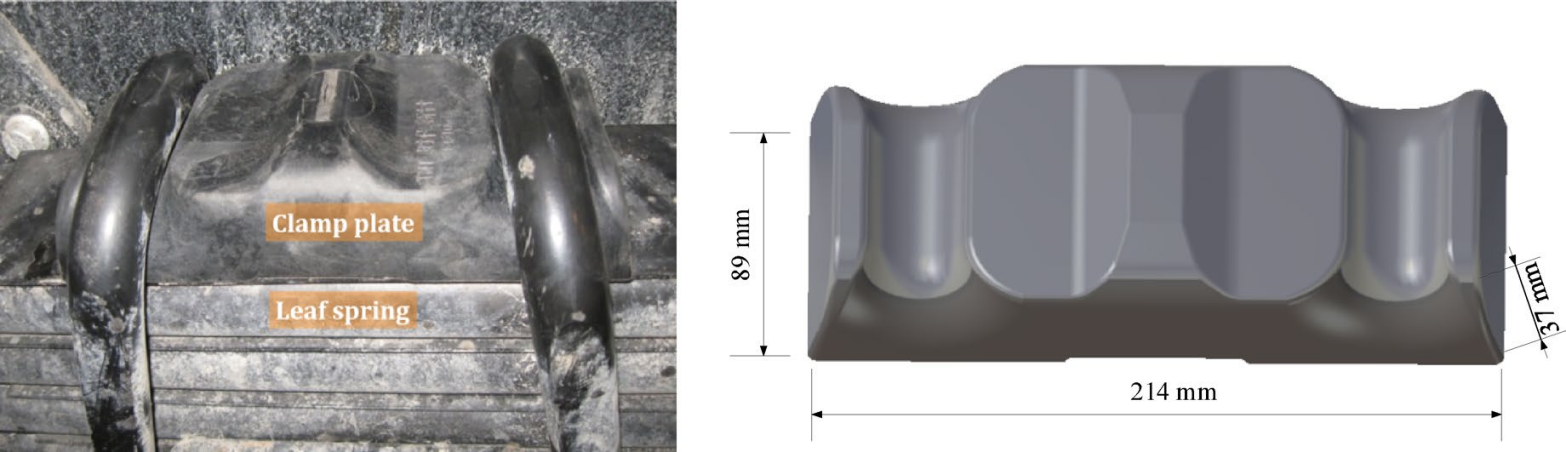
(**a**) Photo of the composite leaf spring clamp plate assembly, (**b**) CAD sketch with profile dimensions of the clamp plate.

The leaf spring clamp plate is a component located on the axle of the vehicle. It is assembly fixed on the axle via a U-bolt, and the axle and the leaf spring jointly play a bearing role. At the same time, the leaf spring assembly is tightened to improve stiffness, thereby augmenting the bearing capacity of the leaf spring. Usually, the leaf spring clamp plate is made of metal materials, such as cast iron and cast steel. Figure 1 shows a photo of the assembly of the metal leaf spring clamp plate. Owing to the high density of metal materials, the clamp plate is relatively heavy, which increases the overall weight of the suspension system and the difficulty of assembly. Glass fiber-reinforced plastics (GFRPs) are optimal composites for leaf spring clamp plates because of their superior mechanical properties, low density, excellent wear resistance, effective vibration and noise attenuation and commendable self-lubrication performance. Figure 2 shows a photo of the assembly of the composite leaf spring clamp plate and the CAD sketch with profile dimensions of the plate.

The original scheme for producing the leaf spring clamp plate was to cast it in ductile iron 450. The quality of the product was high, but the production efficiency was low. To achieve lightweighting and improve production efficiency, injection molding of long glass fiber reinforced thermoplastics (LFT) composed of polyamide-6 (PA6) and 40% glass fiber (GF) was used instead of the original solution.

### Multiscale analysis process

**Fig. 3:**
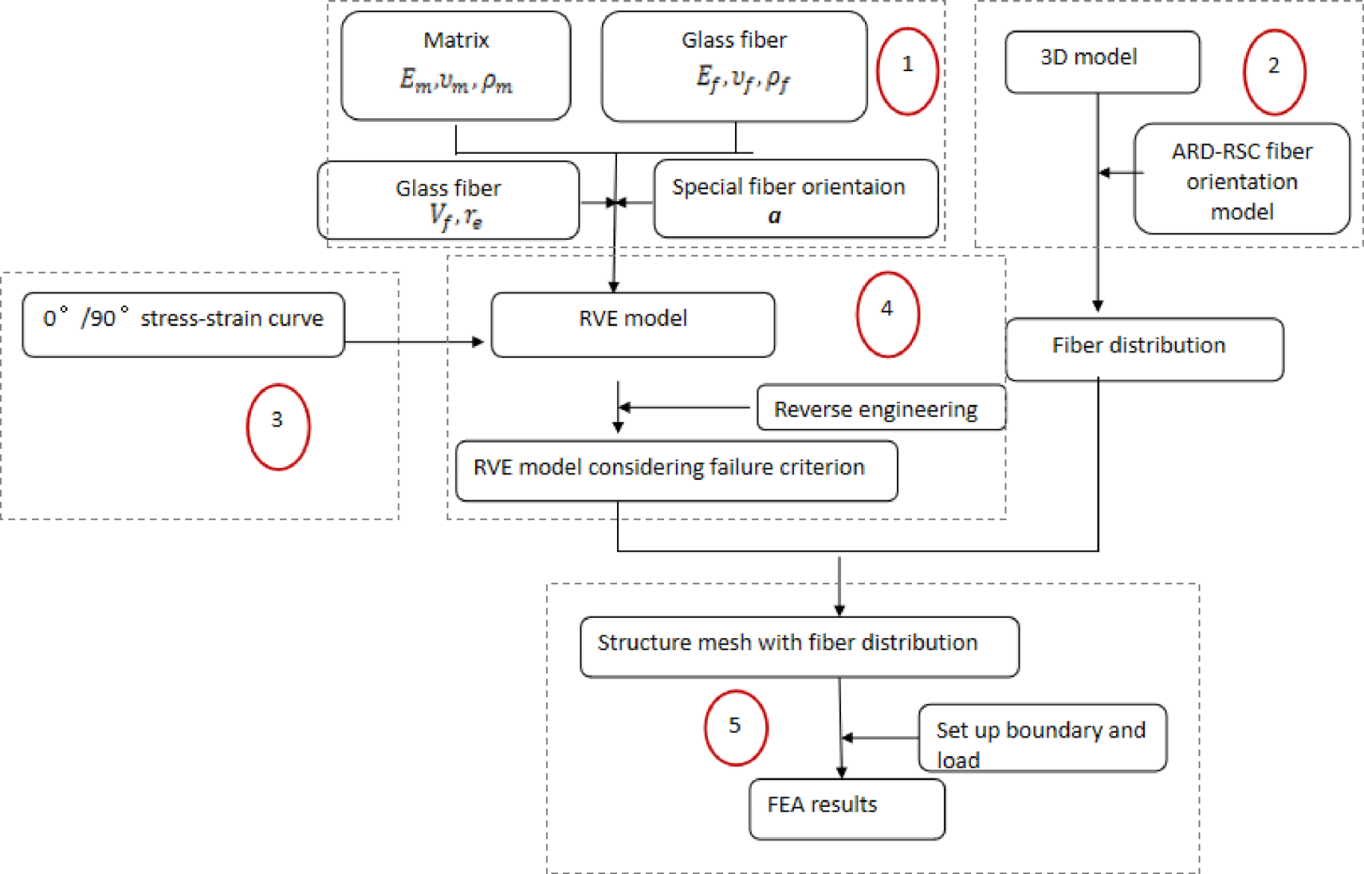
Flowchart of the multiscale FEA analysis cooperating with injection molding process.

The multiscale analysis method combines injection molding and microstructure characterization, accounting for nonaligned fibers and their associated anisotropy, to semi-analytically evaluate the local effective mechanical properties at each point of the GFRP. Microcomputed tomography measurements and injection molding simulations are used to derive the fiber orientation tensor. The two-step mean field homogenization method is applied to determine the mechanical behavior of PA6 + 40%GF (LFT) with distributed-orientation fibers, relying on the fiber orientation tensor. Reverse engineering is employed to acquire the optimal parameters of the matrix. Furthermore, the integral mapping method can facilitate the transformation of the fiber orientation tensor from the injection simulation to the structural simulation model. The detailed analysis procedure is illustrated in Figure 3, which can be divided into the following five steps:

(1) Injection molding simulation of the component using realistic process parameters to determine the fiber orientation distribution.

(2) Preparing test samples with the same material as in the simulation, with varying fiber orientation angles, incorporating at least 0° (flow direction) and 90° (transverse direction) in compliance with the GB/T 1040–2006 standard. Tensile tests are then carried out to obtain the stress‒strain curves for various fiber orientation angles.

(3) Constructing the mesoscopic representative volume element (RVE) model. The theoretical stress‒strain curves are derived from the RVE models according to the specified properties of the PA6 and the glass fiber. The glass fiber is defined as a homogeneous and isotropic linear elastic material, whereas the matrix PA6 is assumed to be a homogeneous and isotropic elastoplastic material and is modeled by the J_2_-plasticity theory.

(4) Identifying the RVE model parameters via reverse engineering. The mesoscopic RVE model of the composite material is reverse engineered using the experimentally obtained stress‒strain curves for various fiber orientation angles, and the matrix parameters are adjusted to ensure that the theoretically derived stress‒strain curves in designated directions align with the experimental curves.

(5) Mesh mapping of the fiber orientation information from the injection molding simulation to the structural analysis. The mechanical simulations are finally performed using appropriate boundary conditions and the material model built in Steps 3 and 4 to analyze the stress and strain fields in the part.

## Thermoplastic injection molding simulation

The thermoplastic injection molding simulation was executed using the commercial software Autodesk MoldFlow, which can analyze the entire injection process. The filling process is proven to substantially influence the fiber orientation distribution; thus, by analyzing the product molding process, the fiber orientation distribution within the product can be derived, represented as a fiber orientation tensor.

### Fiber orientation model

Compared with short glass fiber-reinforced composites, the leaf spring clamp plate is made of PA6 + 40% GF (LFT), which exhibits greater interactions among long glass fibers. The cross-sectional core layer becomes wider; that is, the region where the fiber orientation deviates from the melt flow direction increases in width^18^. Simultaneously, elongated long glass fibers break during the injection procedure.

The fiber orientation state is described by the following second and fourth-order orientation tensors^19^:

1 $${\mathbf{A}}=\int\limits_{{\left\| {\mathbf{p}} \right\|=1}} {{\mathbf{pp}}\varphi \left( {\mathbf{p}} \right){\text{d}}{\mathbf{p}}},{\mathbb{A}}=\int\limits_{{\left\| {\mathbf{p}} \right\|=1}} {{\mathbf{pppp}}\varphi \left( {\mathbf{p}} \right){\text{d}}{\mathbf{p}}}$$

where $\varphi \left( {\mathbf{p}} \right)$ is the fiber orientation distribution function defining the fiber volume fraction in the direction of the unit vector ${\mathbf{p}}$ along the fiber axis. The integration is conducted over the unit sphere.

The present analysis employs the anisotropic rotary diffusion (ARD) - reduced strain closure (RSC) fiber orientation model^20,21^, as defined in Eq. (2). This model, introduced by Phelps and Tucker^20^ in 2009, is specifically designed for LFT, incorporating ARD through the replacement of the interaction coefficient *C*_I_ by an ARD tensor and accounting for the effects of fiber fracture^21^.

2 $${\mathbf{\dot {A}}}=\left( {{\mathbf{W}} \cdot {\mathbf{A}} - {\mathbf{A}} \cdot {\mathbf{W}}} \right)+\xi \left( {{\mathbf{D}} \cdot {\mathbf{A}}+{\mathbf{A}} \cdot {\mathbf{D}} - 2{\mathbb{A}}{\text{:}}{\mathbf{D}}} \right)+\dot {\gamma }\left[ {2{\mathbf{C}} - 2{\text{tr}}\left( {\mathbf{C}} \right){\mathbf{A}} - 5\left( {{\mathbf{C}} \cdot {\mathbf{A}}+{\mathbf{A}} \cdot {\mathbf{C}}} \right)+10{\mathbb{A}}{\text{:}}{\mathbf{C}}} \right]$$

where **W**: the vorticity tensor, ${\mathbf{W}}=\frac{1}{2}\left( {\nabla {\mathbf{u}} - \nabla {{\mathbf{u}}^T}} \right)$ with flow gradient $\nabla {\mathbf{u}}$; **D**: the rate of strain tensor, ${\mathbf{D}}=\frac{1}{2}\left( {\nabla {\mathbf{u}}+\nabla {{\mathbf{u}}^T}} \right)$; $\dot {\gamma }$: the magnitude of the rate of strain tensor, $\dot {\gamma }=\sqrt {2{\mathbf{D}}:{\mathbf{D}}}$; $\xi$: the fiber shape function, $\xi =\frac{{a_{r}^{2} - 1}}{{1+a_{r}^{2}}}$ with fiber aspect ratio ${a_r}=\frac{L}{d}$ with fiber length *L* and fiber diameter *d*; and ${\mathbf{C}}$: rotary diffusion tensor, ${\mathbf{C}}={b_1}{\mathbf{I}}+{b_2}{\mathbf{A}}+{b_3}{{\mathbf{A}}^2}+\frac{{{b_4}}}{{\dot {\gamma }}}{\mathbf{D}}+\frac{{{b_5}}}{{{{\dot {\gamma }}^2}}}{{\mathbf{D}}^2}$ with ${b_i}\left( {i=1,2, \cdots ,5} \right)$ scalar constants with values obtained through steady-state directional fiber orientation experiments.

### Fiber orientation tensor

**Fig. 4:**
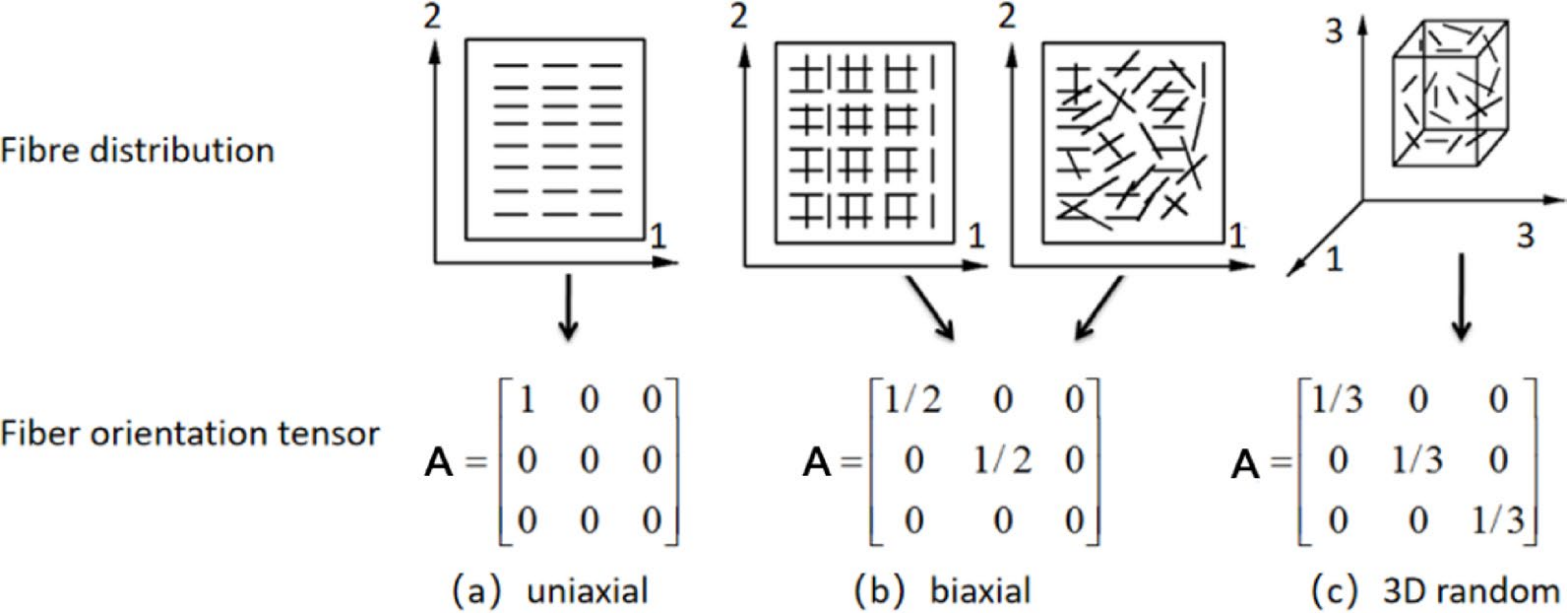
Fiber orientation tensor.

For short/long glass-fiber reinforced composites, the fiber orientation distribution is generally characterized by the fiber orientation tensor^22–24^. Upon diagonalization, the second-order tensor presented in Eq. (3) can describe the state of the fiber orientation. Distinct tensors correlate with various orientations of glass fibers, as illustrated in Figure 4, (a) denotes uniaxial orientation, indicating that the fibers are arranged along a single principal axis; (b) refers to biaxial orientation, wherein the fibers are uniformly distributed along two principal axes, but may also imply random orientation within the plane; (c) signifies a distribution that is uniformly aligned along three principal axes, and may also suggest random orientation in three-dimensional space.

3 $$\mathbf{A}=\sum\limits_{i} {{\lambda _i}\left( {{{\mathbf{e}}_i} \otimes {{\mathbf{e}}_i}} \right)}\,with\,{\lambda _1}+{\lambda _2}+{\lambda _3}=1$$

**Fig. 5:**
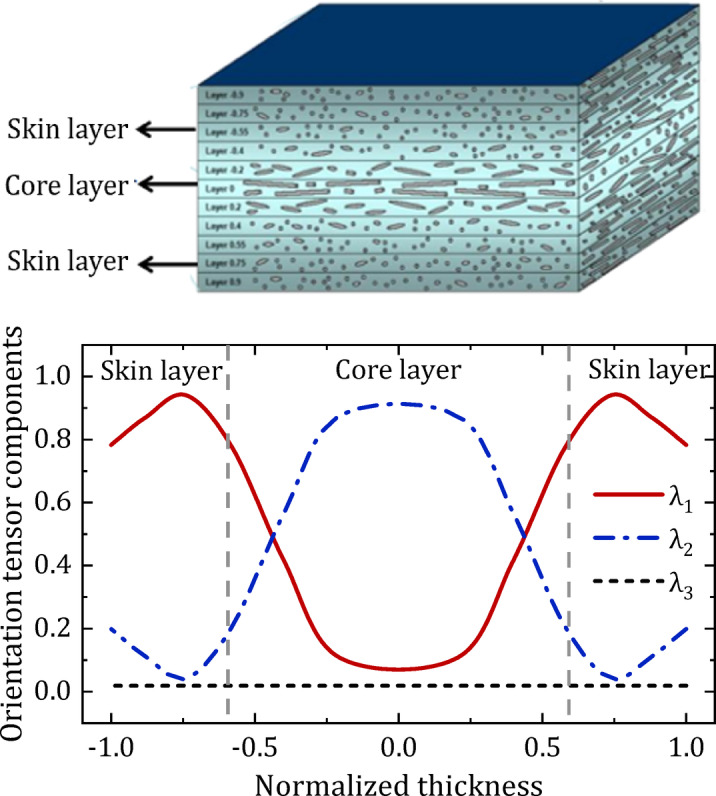
Three components of the fiber orientation tensor along the sample normalized thickness.

The mesh for microstructure reconstruction was defined such that the three principal fiber orientation tensor components broadly correspond to the directions of the Cartesian coordinates: ${\lambda _1}$ corresponds to the fiber orientation in the X direction (flow direction), ${\lambda _2}$in the Y direction (transverse to flow), and ${\lambda _3}$ in the Z direction (thickness direction). Figure 5 shows the simulated results of the fiber orientation distribution of the molded sample panel in the thickness direction. The horizontal axis represents the normalized thickness (± 1.0 indicates the cavity wall surface and 0 indicates the cavity center). The vertical axis represents the value of the components ${\lambda _1}$, ${\lambda _2}$, and ${\lambda _3}$.

### Fiber orientation analysis

Fiber orientation distribution is obtained by Moldflow simulation. The injection molding process parameters for the simulation are consistent with those of the actual injection molding process, and are given in Table 1. In the simulation, the injection filling time is 10 s. When the filling volume reaches 95%, the filling phase is switched to the packing phase with a pressure of 10 MPa, and the packing duration was 10 s.

**Table 1:** Process parameters for the injection molding simulation.

Parameter	Value
Melt density (g/cm^3^)	1.40
Solid density (g/cm^3^)	1.5115
Ejection temperature (℃)	182
Absolute maximum melt temperature (℃)	300
Melt temperature (℃)	260
Mold temperature (℃)	90
Injection time (s)	10
V/P switch-over (%)	95
Packing duration (s)	10
Packing pressure (MPa)	10

**Fig. 6:**
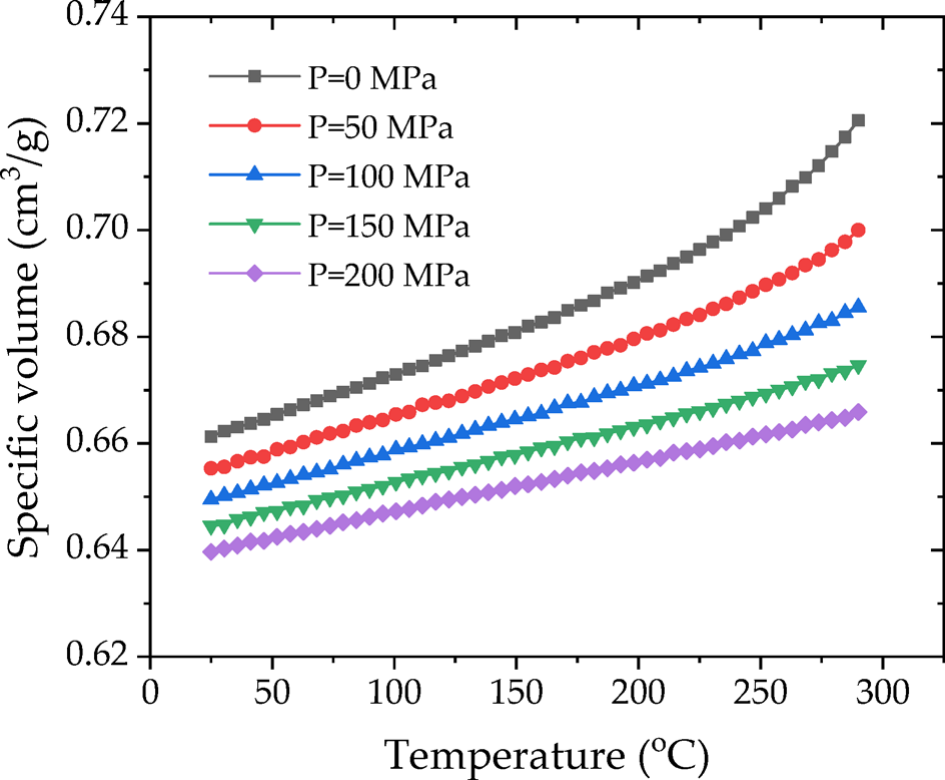
PVT curves.

The PVT curves of the FRP composites investigated in this study are given in Figure 6. This specific volume dependency on the pressure and temperature of the material is modeled by the two-domain Tait equation of state^25^, the typical model used in injection molding simulation, as given below:

4 $$v\left( {T,p} \right)={v_0}\left( T \right)\left[ {1 - C\ln \left( {1+\frac{p}{{B\left( T \right)}}} \right)} \right]+{v_t}\left( {T,p} \right)$$

where, $v\left( {T,p} \right)$ is the specific volume at a given temperature and pressure, ${v_0}$ is the specific volume at zero gauge pressure, *T* is the temperature in [K], *p* is the pressure in [Pa], *C* is a universal constant of 0.0894, and *B* accounts for the pressure sensitivity of the material and is defined below.

5 $${v_0}\left( T \right)=\left\{ {\begin{array}{*{20}{c}} {{b_{{\text{1m}}}}+{b_{{\text{2m}}}}\left( {T - {b_{\text{5}}}} \right),{\text{ for }}T{\text{>}}{T_{\text{t}}}} \\ {{b_{{\text{1s}}}}+{b_{{\text{2s}}}}\left( {T - {b_5}} \right),{\text{ for }}T{\text{<}}{T_{\text{t}}}} \end{array}} \right.$$

6 $$B\left( T \right)=\left\{ {\begin{array}{*{20}{c}} {{b_{{\text{3m}}}}\exp \left[ { - {b_{{\text{4m}}}}\left( {T - {b_{\text{5}}}} \right)} \right],{\text{ for }}T{\text{>}}{T_{\text{t}}}} \\ {{b_{{\text{3s}}}}\exp \left[ { - {b_{{\text{4s}}}}\left( {T - {b_{\text{5}}}} \right)} \right],{\text{ for }}T{\text{<}}{T_{\text{t}}}} \end{array}} \right.$$

7 $${v_t}\left( {T,p} \right)=\left\{ \begin{gathered} 0,{\text{ for }}T{\text{>}}{T_{\text{t}}} \hfill \\ {b_{\text{7}}}\exp \left[ {{b_{\text{8}}}\left( {T - {b_{\text{5}}}} \right) - {b_9}p} \right],{\text{ for }}T{\text{<}}{T_{\text{t}}} \hfill \\ \end{gathered} \right.$$

8 $${T_t}\left( p \right)={b_5}+{b_6}p$$

in which ${T_{\text{t}}}$ represents the volumetric transition temperature, ${b_{{\text{1m}}}}$, ${b_{{\text{2m}}}}$, ${b_{{\text{3m}}}}$, ${b_{{\text{4m}}}}$, ${b_{{\text{1s}}}}$, ${b_{{\text{2s}}}}$, ${b_{{\text{3s}}}}$, ${b_{{\text{4s}}}}$, ${b_{\text{5}}}$, ${b_{\text{6}}}$, ${b_{\text{7}}}$, ${b_{\text{8}}}$ and ${b_{\text{9}}}$ are data-fitted coefficients. The above 13 parameters for the simulation are listed in Table 2.

**Table 2:** Parameters in the two-domain Tait model.

Parameter	Value	Parameter	Value
*b*_1 m_ (m^3^kg^−1^)	0.000711	*b*_1s_ (m^3^kg^−1^)	0.0006977
*b*_2 m_ (m^3^kg^−1^K^−1^)	5.003 × 10^−7^	*b*_2s_ (m^3^kg^−1^K^−1^)	1.46 × 10^−7^
*b*_3 m_ (Pa)	1.47465 × 10^8^	*b*_3s_ (Pa)	2.63427 × 10^8^
*b*_4 m_ (K^−1^)	0.003351	*b*_4s_ (K^−1^)	0.002202
*b*_5_ (K)	544.15	*b*_6_ (KPa^−1^)	2.45 × 10^−7^
*b*_7_ (m^3^kg^−1^)	1.335 × 10^−5^	*b*_8_ (K^−1^)	0.01867
*b*_9_ (Pa^−1^)	1.11 × 10^−8^		

In the mold filling process, the Cross-WLF model is used to describe the temperature, shear rate, and pressure dependency of the FRP melt viscosity^26^:

9 $$\eta =\frac{{{\eta _0}}}{{1+{{\left( {{{{\eta _0}\dot {\gamma }} \mathord{\left/ {\vphantom {{{\eta _0}\dot {\gamma }} {{\tau ^*}}}} \right. \kern-0pt} {{\tau ^*}}}} \right)}^{1 - n}}}}$$

where $\eta$ is the melt viscosity, ${\eta _0}$ is the zero shear viscosity or the ‘Newtonian limit’ in which the viscosity approaches a constant at very low shear rates, $\dot {\gamma }$ is the shear strain rate, ${\tau ^*}$ is the critical stress level at the transition to shear thinning, and *n* is the power law index in the high shear rate regime. The temperature and pressure dependence of the zero shear viscosity is given by the following equation:

10 $${\eta _0}={D_1}\exp \left[ { - \frac{{{A_1}\left( {T - {T_g}} \right)}}{{{A_{\text{2}}}+\left( {T - {T_g}} \right)}}} \right]$$

in which ${T_g}={D_2}+{D_3}p$, ${A_{\text{2}}}={\tilde {A}_2}+{D_3}p$, and ${D_1}$, ${A_1}$, ${\tilde {A}_2}$, ${D_2}$ and ${D_3}$ are data-fitted coefficients.

**Fig. 7:**
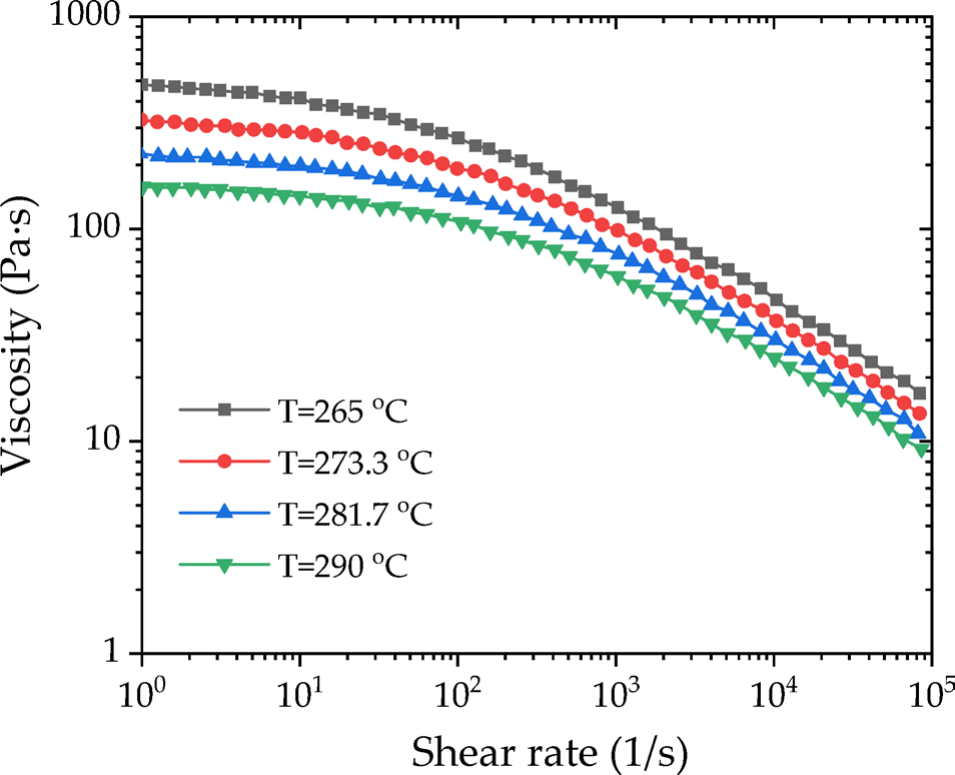
Shear viscosity curves at various temperatures.

The melt viscosity versus shear rate curves for various temperatures of the FRP melt in this study are plotted in Figure 7.

**Fig. 8:**
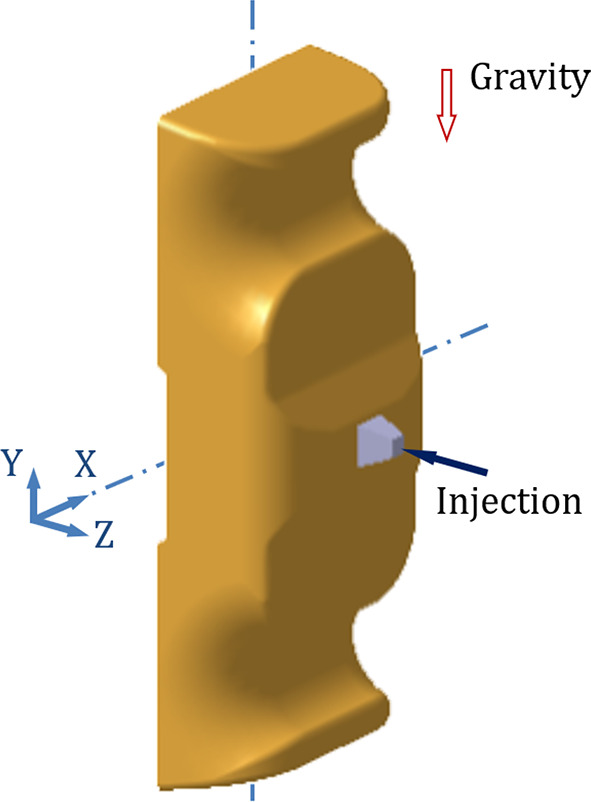
Schematic of vertical injection molding.

**Fig. 9:**
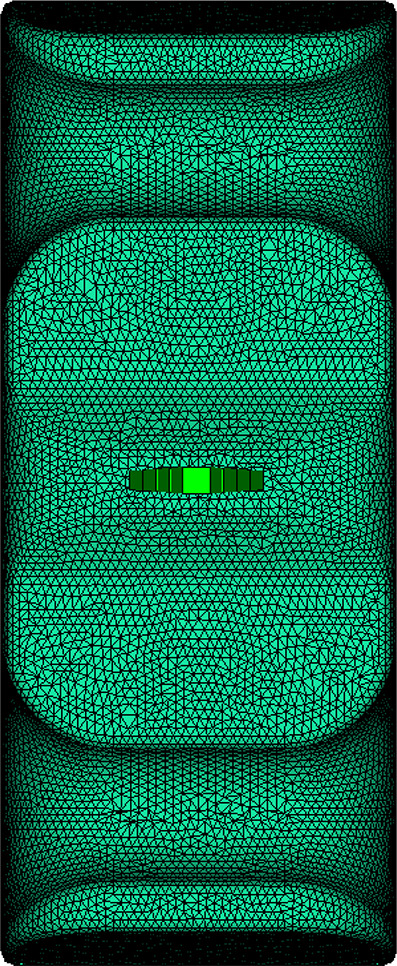
Mesh model.

**Fig. 10:**
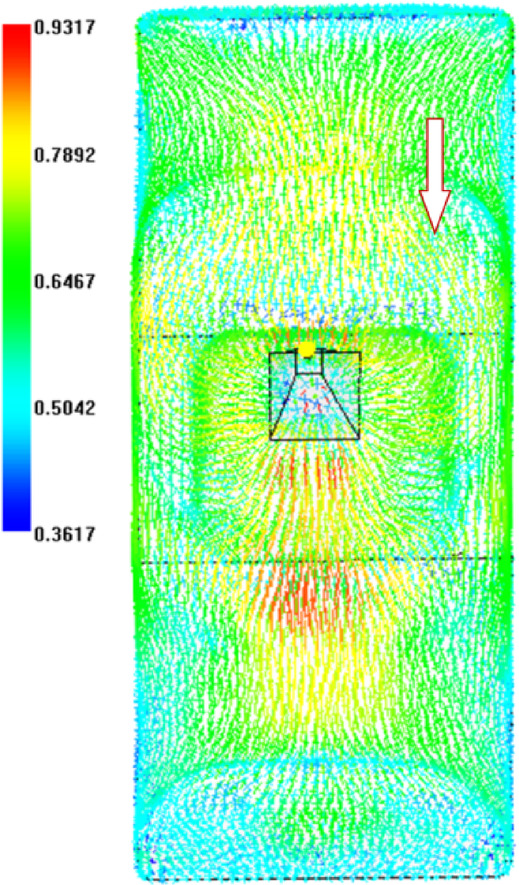
Fiber orientation distributions for vertical injection molding.

For the original production process, the leaf spring clamp plate adopts a cold injection gate positioned at the center. The shape of the gate is a tapered rectangular, with a start width of 6 mm and an end width of 10 mm. The mold was placed vertically (Figure 8), hereinafter referred to as vertical injection molding. Owing to the thick wall of the product, the influence of gravity must be considered. The injection molding analysis is carried out via Autodesk MoldFlow. The mesh configuration consists of tetrahedral elements, as shown in Figure 9. The fiber orientation distribution was derived through process analysis, as shown in Figure 10, where the arrow indicates the direction of gravity. The analysis results show that the fiber orientation consistency near the ground is satisfactory; however, the consistency of the fiber orientation on the side opposite the ground is comparatively poor.

## Meso-constitutive model

The meso-constitutive model of the composite is constructed on the basis of the Mori–Tanaka theory.

### Mori–Tanaka model

Many fundamental and pioneering studies on the meso-constitutive modeling of glass fiber-reinforced composites have been performed by numerous researchers^27–33^. In 1973, Mori and Tanaka^33^ proposed a method named after their names. The method can effectively determine the elastic modulus of multiphase heterogeneous materials based on the Eshelby’s equivalent inclusion theory^28^, taking into account the interactions induced by the work hardening of dispersed materials in multiphase composites.

The basic idea of Mori–Tanaka’s mean-field method is to encapsulate the inclusion constituents within the original matrix and apply the average matrix strains in the RVE as the far field strain, thus facilitating the mapping of the interactions between inclusions and determining the equivalent elasticity coefficients of fiber-reinforced composites for a variety of nonhomogeneous inclusion scenarios.

Let *f* be the fiber volume fraction, and let ${\mathbf{E}_0}$ and ${\mathbf{E}_{^{1}}}$ be the fourth order stiffness tensors of the matrix and the fibers, respectively. The effective stiffness tensor $\mathbf{E}$ of the composite is given by

11 $$\mathbf{E}={\mathbf{E}_0}+f\left( {{\mathbf{E}_1} - {\mathbf{E}_0}} \right)\mathbf{B}$$

where $\mathbf{B}$ denotes the Mori-Tanaka fiber strain concentration tensor.

12 $$\mathbf{B}=\mathbf{T}{\left[ {\left( {1 - f} \right)\mathbf{I}+f\mathbf{T}} \right]^{ - 1}}$$

and

13 $$\mathbf{T}={\left[ {\mathbf{I}+\mathbf{S}\mathbf{E}_{0}^{{ - 1}}\left( {{\mathbf{E}_1} - {\mathbf{E}_0}} \right)} \right]^{ - 1}}$$

in which $\mathbf{I}$ is the fourth-order unit tensor, and **S** is the Eshelby tensor^28^. The tensor **T** correlates the strain tensor ${\varvec{\upvarepsilon}_1}$ inside the fiber and the matrix uniform strain ${\varvec{\upvarepsilon}_0}$ subjected at infinity as follows

14 $${\varvec{\upvarepsilon}_1}=\mathbf{T}{\varvec{\upvarepsilon}_0}$$

### *J*_2_*-Plasticity model*

The construction of the mesoscale constitutive model based on Mori–Tanaka theory requires the input of material parameters for the matrix and reinforcement phases separately. In the present analysis, the glass fiber is assumed to be homogeneous and isotropic linear elastic, whereas the matrix PA6 is considered a homogeneous and isotropic elastoplastic material^34–38^.

The stress-strain curve of the matrix is fitted via the J_2_-plasticity model, which is given by Eq. (15).

15 $$\left\{ \begin{gathered} {\mathbf{\sigma }}=\mathbf{E}:{\varvec{\upvarepsilon}^{\text{e}}}\begin{array}{*{20}{c}} ,&{{\text{if }}{\sigma _{{\text{eq}}}} \leqslant {\sigma _{\text{Y}}}} \end{array} \hfill \\ {\sigma _{{\text{eq}}}}={\sigma _{\text{Y}}}+kq+{R_\infty }\left( {1 - {e^{ - mq}}} \right)\begin{array}{*{20}{c}} ,&{{\text{if }}{\sigma _{{\text{eq}}}}>{\sigma _{\text{Y}}}} \end{array} \hfill \\ \end{gathered} \right.$$

where ${{\mathbf{\varepsilon }}^e}$ is the elastic strain, ${\sigma _{\text{Y}}}$ is the initial yield stress, and $k$,${R_\infty }$, and $m$ represent the linear hardening modulus, hardening modulus, and hardening exponent, respectively. The parameter $q$ represents the accumulated plastic strain, and it is expressed as:

16 $$q=\int_{0}^{t} {\dot {q}\left( \tau \right){\text{d}}\tau }\,with\,\dot {q}=\sqrt {\frac{2}{3}{{{\mathbf{\dot {\varepsilon }}}}^{\text{p}}}:{{{\mathbf{\dot {\varepsilon }}}}^{\text{p}}}}$$

where ${{\mathbf{\dot {\varepsilon }}}^{\text{p}}}$ is the plastic strain rate, and ${\sigma _{{\text{eq}}}}$ in Eq. (15) denotes the von Mises equivalent stress, which is defined by the second invariant of the deviatoric stress tensor **s**.

17 $${\sigma _{{\text{eq}}}}=\sqrt {3{J_2}\left( {\mathbf{s}} \right)} =\sqrt {\tfrac{3}{2}{\mathbf{s}}:{\mathbf{s}}}$$

The material parameters of the matrix and fiber applied to the simulations in this study are listed in Table 3.

**Table 3:** Basic material parameters for the matrix and fibers.

	Matrix	Fiber
Density (ton/mm^3^)	1.14 × 10^−9^	2.54 × 10^−9^
Young’s modulus (MPa)	3000	72,000
Poisson’s ratio	0.37	0.22
Initial yield stress, ${\sigma _Y}$(MPa)	20	/
Hardening modulus, ${R_\infty }$ (MPa)	30	/
Hardening exponent, *m*	75	/
Linear hardening modulus, *k* (MPa)	10	/

### Sample tests with different fiber orientations

The mechanics-based constitutive model of composites considers the perfect combination of matrix and reinforcement phases and ignores the impact of defects resulting from the actual injection molding process. To ensure that the constitutive model actually represents the true mechanical response of the composites, it is necessary to calibrate the model’s material parameters via reverse engineering using actual test curves.

**Fig. 11:**
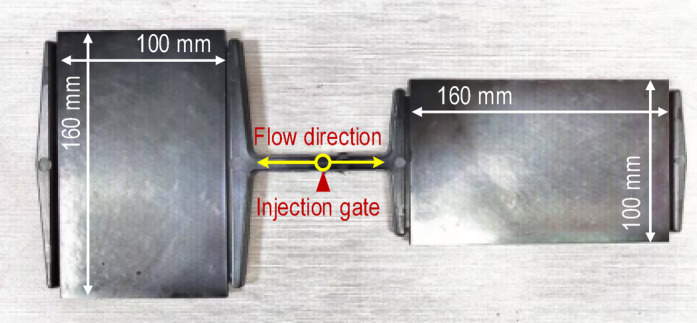
Injection molded sample panels.

**Fig. 12:**
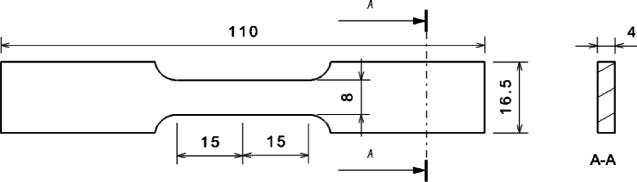
Dimensions of the specimen.

**Fig. 13:**
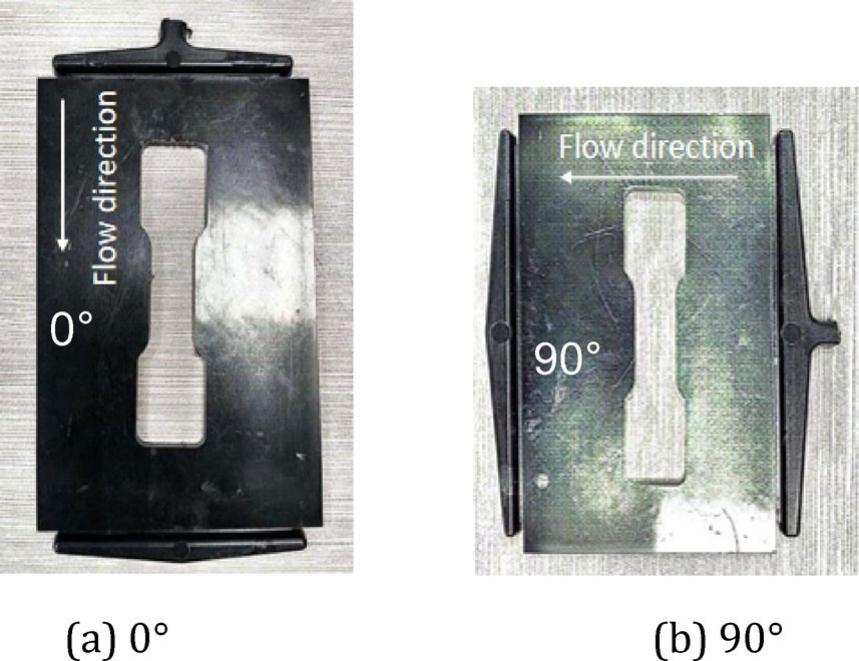
Sample panels after specimen cutting-out, showing specimens with 0° and 90° fiber orientations.

**Fig. 14:**
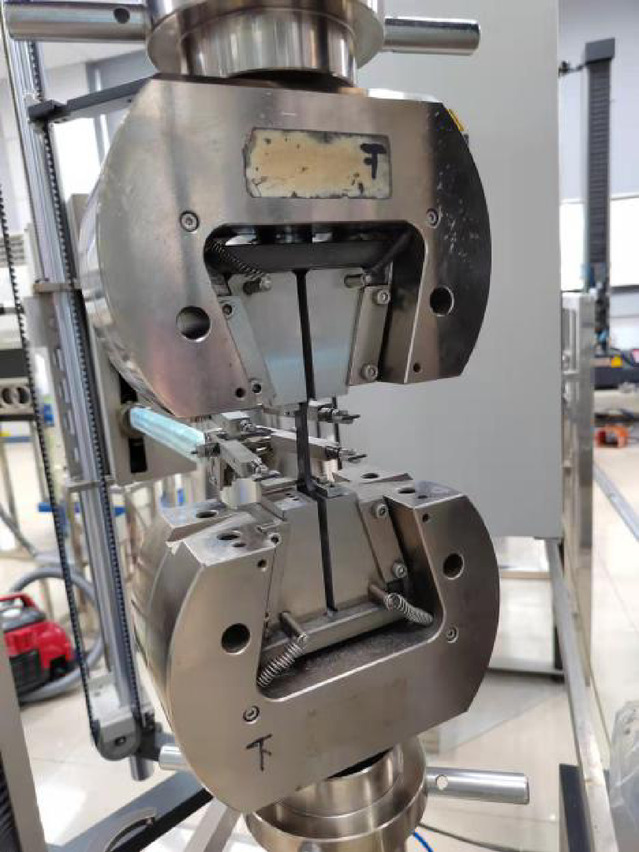
Tensile test machine.

Sample panels with varying fiber orientations were injection molded using the identical materials of PA6 + 40% GF (long glass fiber) with the leaf spring clamp plate, as shown in Figure 11. Haitian injection molding machine with a clamping force of 470 tons and a maximum injection volume of 1200 ml was used to make the sample panels. The material was dried at 100 °C for 4 h, the melt temperature was 260 °C, and the mold temperature was 90 °C. The filling time was set to 10 s. When the filling volume reached 95%, the filling process was switched to a pressure-holding process with a pressure of 10 MPa, and the pressure-holding duration was 10 s. The average cycle time of the whole injection molding was approximately 150 s, which included the mold clamping time, injection time, pressure-holding time, and cooling time. The sample panel dimensions were 160 mm × 100 mm × 4 mm, and the specimens with different fiber orientations were extracted from the panels. The melt flow direction is designated 0°, whereas the direction perpendicular to the melt flow direction is designated 90°. The dimensions of the specimen are illustrated in Figure 12. For reverse engineering, it is advisable to use a minimum of two specimen sets with fiber orientation angles of 0° and 90°, as shown in Figure 13. Tensile tests were performed at room temperature and 50% relative humidity in accordance with the GB/T 1040–2006 standard on a Zwick/Roell universal tensile testing machine Z020 equipped with a 20 kN force cell, and the strains were measured using an extensometer with a gauge length of 25 mm. The test machine fixture with the specimen loaded is shown in Figure 14. All tests were carried out at a constant crosshead speed of 1.0 mm/min, stress-strain curves were obtained for the 0° and 90° specimens.

### Reverse engineering for model parameters

**Fig. 15:**
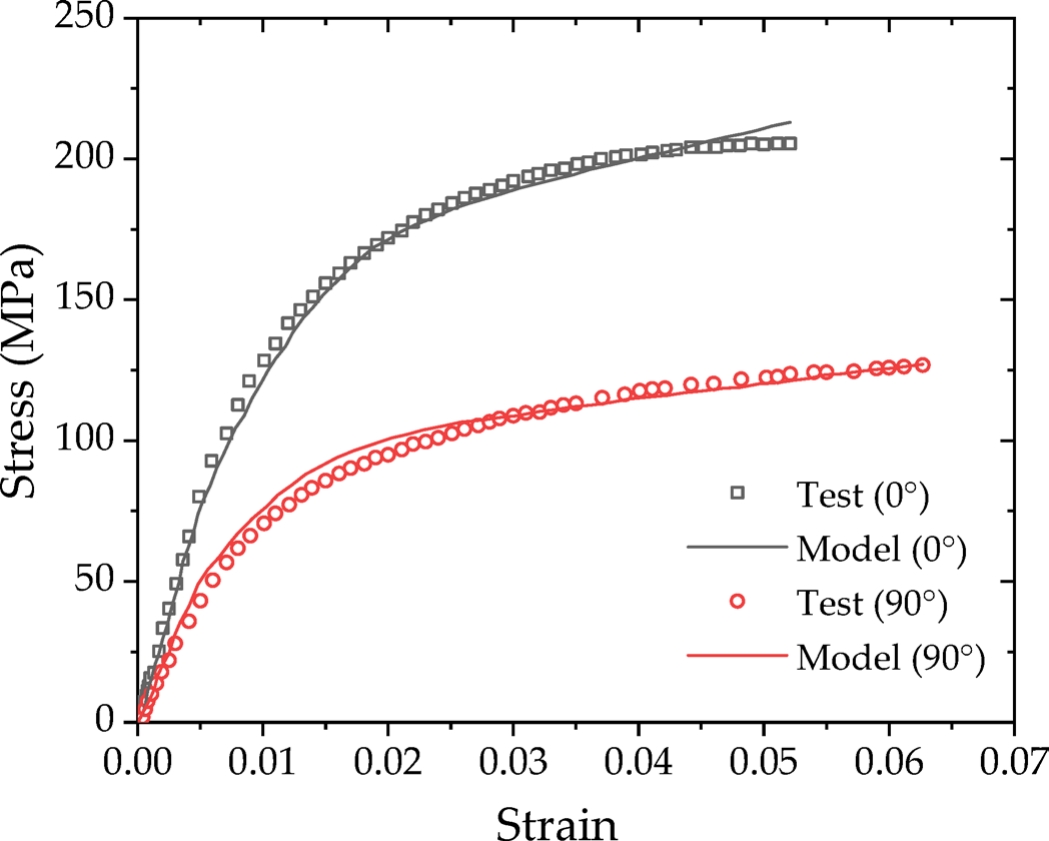
Comparison of the model predictions with the experimental curves.

The computed stress‒strain curves are derived from the properties of the matrix and fibers as well as the fiber geometry and their spatial distribution within the matrix, showing a certain deviation from the experimental curves. To minimize calculation deviation, a reverse engineering approach is employed to iteratively optimize the elastic modulus, yield strength, fiber aspect ratio and other parameters. Reverse engineering in an iterative optimization framework aims to determine the ideal values of design variables (i.e., material model parameters) that reduce the discrepancies between model predictions and experimental measurements. The revised parameters for the meso-constitutive model are presented in Table 4. The tensile strength of the material with a fiber orientation angle of 0° was 205 MPa. This study employed the maximum stress criterion to determine the material’s failure. Figure 15 shows that the model predictions are in good agreement with the measured stress-strain curves.

**Table 4:** Material parameters of the matrix and fibers.

	Matrix	Fibers
Density(ton/mm^3^)	1.14 × 10^−9^	2.54 × 10^−9^
Young’s modulus (MPa)	4510	72,000
Poisson coefficient	0.41	0.22
Yield stress (MPa)	30	/
Hardening modulus (MPa)	25	/
Hardening exponent	145	/
Linear hardening modulus (MPa)	150	/

## Finite element strength analysis

### Mesh mapping

**Fig. 16:**
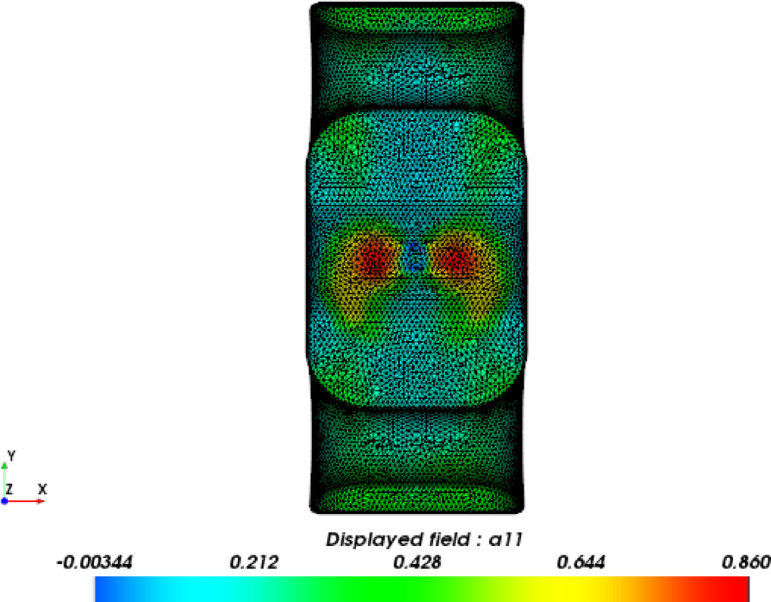
Fiber orientation distribution based on the molding analysis mesh.

**Fig. 17:**
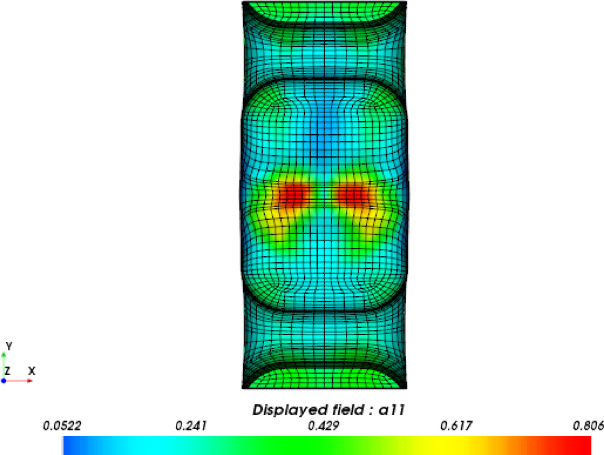
Fiber orientation distribution based on the strength analysis mesh.

**Fig. 18:**
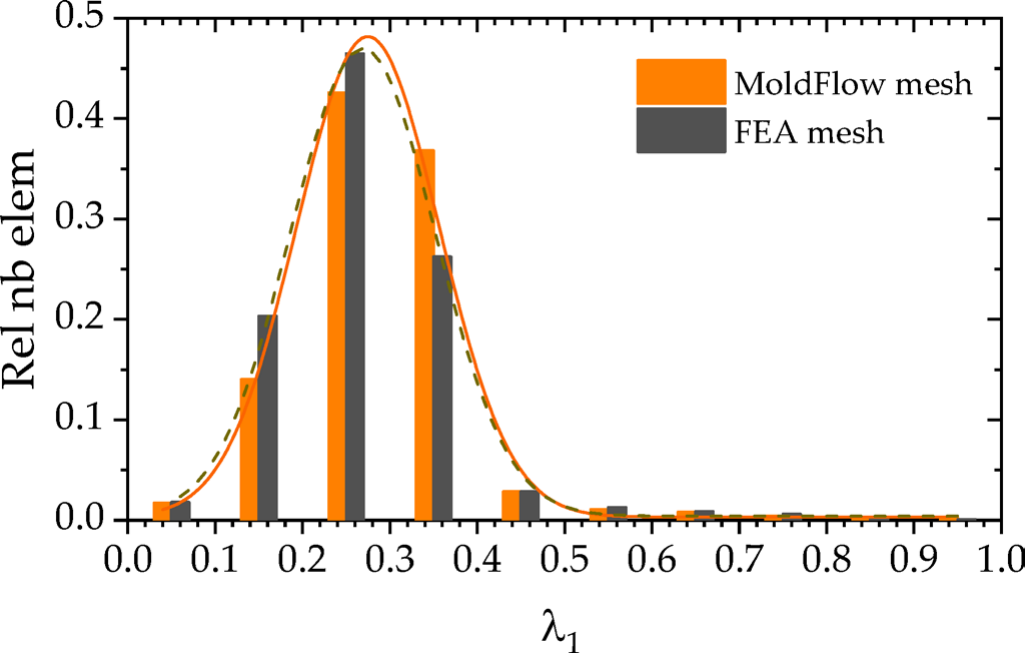
Relative number of elements from global error indicator for mesh mapping.

The structure mesh for finite element analysis (FEA) is usually different from the mesh used to simulate the injection process. The fiber orientation distribution data (Figure 16) acquired from the injection molding simulation were mapped to the structural mesh (Figure 17) using the integration point-to-integration point mapping method. A global error indicator was monitored to assess the difference in fiber orientation between Moldflow analysis and FEA analysis. As shown in Figure 18, the solid line represents the relative number of elements for the principal component λ_1_ of the fiber orientation tensor in the Moldflow analysis, whereas the dashed line represents that in the FEA analysis; thus, the distributions of the relative number of elements before and after mesh mapping are essentially identical, ensuring that the stresses predicted via FEA strength analysis are not significantly affected by mesh mapping.

### Boundary conditions

**Fig. 19:**
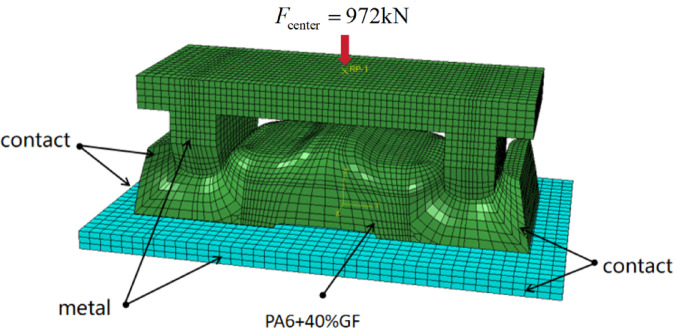
Finite element analysis model.

In practice, the leaf spring clamp plate is a critical component in automotive chassis systems; it is connected to the axle via U-bolts. Under proper installation conditions, the preloading force is applied only by means of U-bolts, each set of which consists of two screws with a diameter of 24 mm, forming a specific tightening torque of 700 N·m, which is the required value for structural integrity. On the basis of the relationship between the tightening torque and the bolt preload given in Eq. (18), the preload force applied to a single screw is calculated to be 243 kN. Thus, the combined preload applied to the clamp plate by each U-bolt is 486 kN. There are two U-bolts in the leaf spring clamp plate assembly; thus, the total force exerted by the U-bolts on the clamp plate is 972 kN. In the FEA simulation, a concentrated force of 972 kN is applied at the center of the top geometry; this force is transferred to the clamp plate through the contact between the semicircular head of the top geometry and the saddle of the clamp plate, as shown in Figure 19. The top geometry is made of steel, which is much stiffer than the clamp plate, therefore, the contact stress at each saddle of the clamp plate has a combined force of 486 kN in the vertical direction because of the perfect match between the semicircular head of the top geometry and the saddle of the clamp plate.

18 $$M=kFD$$

where *M* is the tightening torque, *F* is the preload in each screw of the U-bolt, *k* is a coefficient with value of 0.12, and *D* is the screw diameter.

### Finite element analysis

**Fig. 20:**
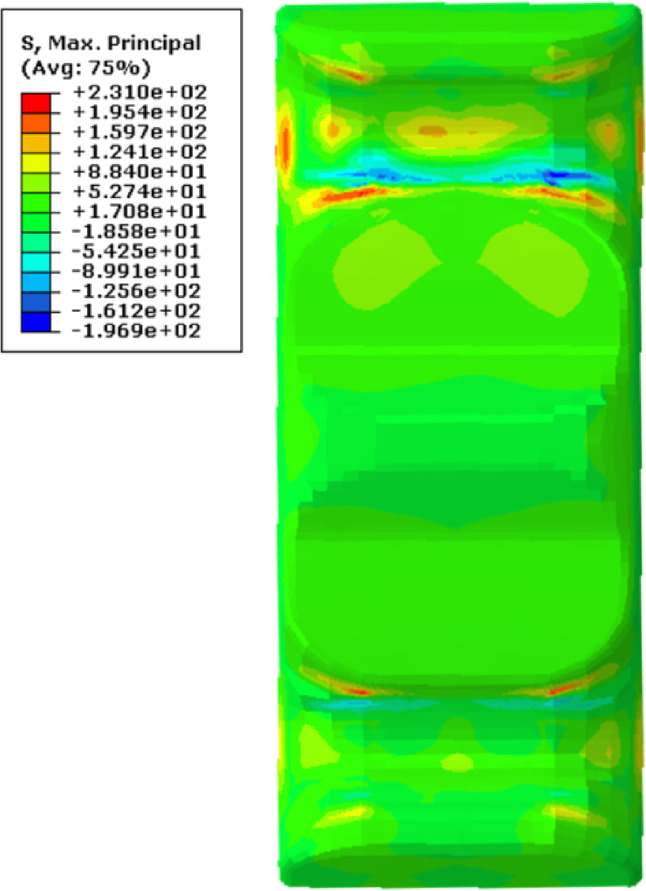
Stress field via FEA in the vertically injection-molded leaf spring clamp plate.

A macroscopic structural stress analysis was performed to derive the stress field via the mapped fiber orientation distribution depicted in Figure 17 and the constitutive model of the composite obtained in Sect. 4. As shown in Figure 20, the maximum principal stress in the leaf spring clamp plate is 231 MPa, which is located at the upper end of the plate when it is vertically injection molded, and is higher than the material’s allowable tensile stress of 205 MPa, leading to fracture. In contrast, the stress at the lower end is approximately 120 MPa. Despite the geometrical symmetry, the stress distribution within the plate is markedly disparate.

### Sample failure analysis

**Fig. 21:**
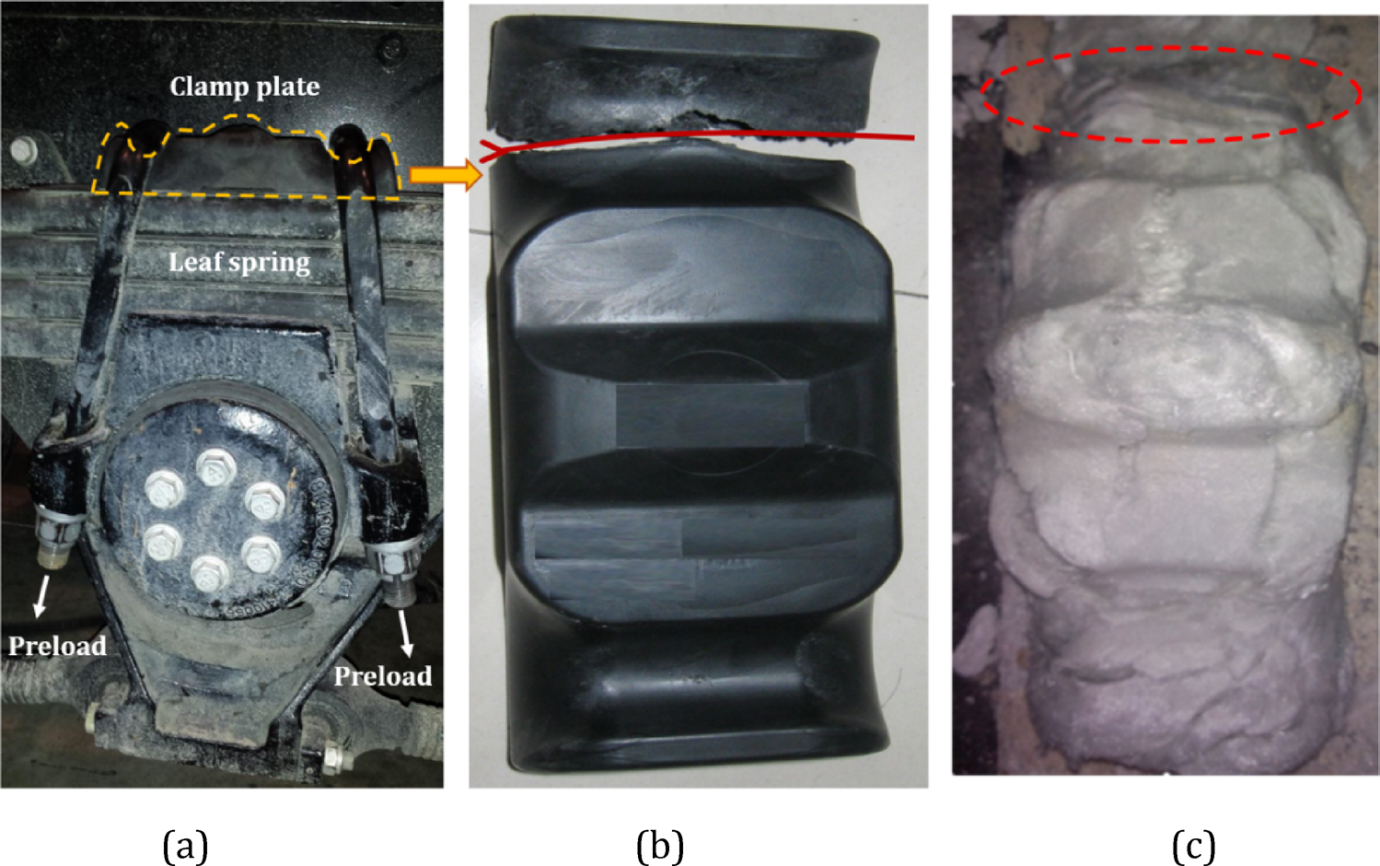
Failure test in actual car installation (a), broken sample (b) and sintered sample (c).

Two groups of vertically injection-molded leaf spring clamp plates were used for testing and failure analysis. As illustrated in Figure 21(a), the first group was subjected to a load of 972 kN (4 × 243 kN) using a torque wrench with a capacity of 1000 N·m, following the specified tightening procedure:

(1) Initial positioning and pre-installation. Mount two sets of U-bolts at designated locations, and thereafter affix four matching nuts. Manually secure the nuts until they make complete contact with the clamp plate surface and are immovable by hand, so achieving the initial location of the clamp plate and eliminating assembly gaps.

(2) Diagonal cross pre-tightening​. Sequentially pre-tighten the nuts in a diagonal cross pattern and apply ​50% of the specified torque (i.e., 350 N·m)​​ with the torque wrench. Ensure a ​consistent rotational angular velocity of 10°–20°/s while tightening to guarantee uniform stress distribution across nuts and prevent clamp plate misalignment or localized gasket deformation.

(3) Incremental torque adjustment to the specified value​. Preserve the diagonal cross sequence while progressively increasing torque to the specified value of 700 N·m in three phases: Initially, augment the torque by ​20% of the specified torque (i.e., 140 N·m)​, culminating a total of ​490 N·m. Subsequently, augment the torque by another ​20% of the specified torque (i.e., 140 N·m)​, achieving a total of ​630 N·m, and ultimately, increase the torque by ​10% of the specified torque (i.e., 70 N·m)​, culminating in a final torque of ​700 N·m. Following each phase, examine the uniformity of flange gaps and the condition of nut contact to ensure there is no loosening or biased loading.

Upon the total torque of 700 N·m, the clamp plate exhibited fracture, with the fracture location aligning with the findings in the previously mentioned multiscale FEA, as depicted in Figure 21(b).

To determine whether the fracture resulted from the uneven fiber distribution, the second group was sintered to eliminate the matrix resin and retain only the fibers, after which the fiber distribution was examined. Figure 21(c) demonstrates that the fracture region had a lower density of glass fibers and a more diverse orientation than the other regions did. It can be concluded that the fiber orientation distribution is responsible for the local stress concentration and triggers fracture failure.

## Optimization scheme

The uneven distribution of fiber orientation induces stress concentration, resulting in fracture failure. Consequently, to enhance the strength of the injection-molded product, minimizing the disparities in the fiber orientation distribution arising from the molding process is essential. Two aspects were optimized in this study: altering the injection direction from vertical to horizontal and optimizing the plate wall thickness.

### Altering the injection molding direction

**Fig. 22:**
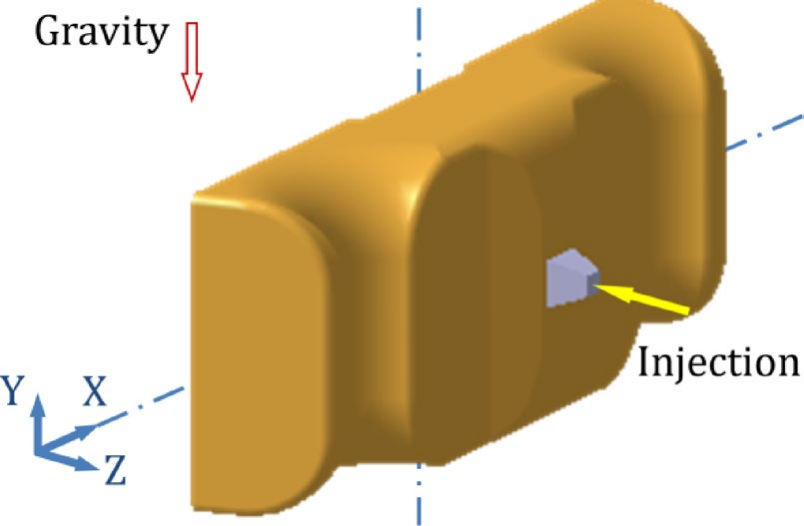
Schematic of horizontal injection molding.

**Fig. 23:**
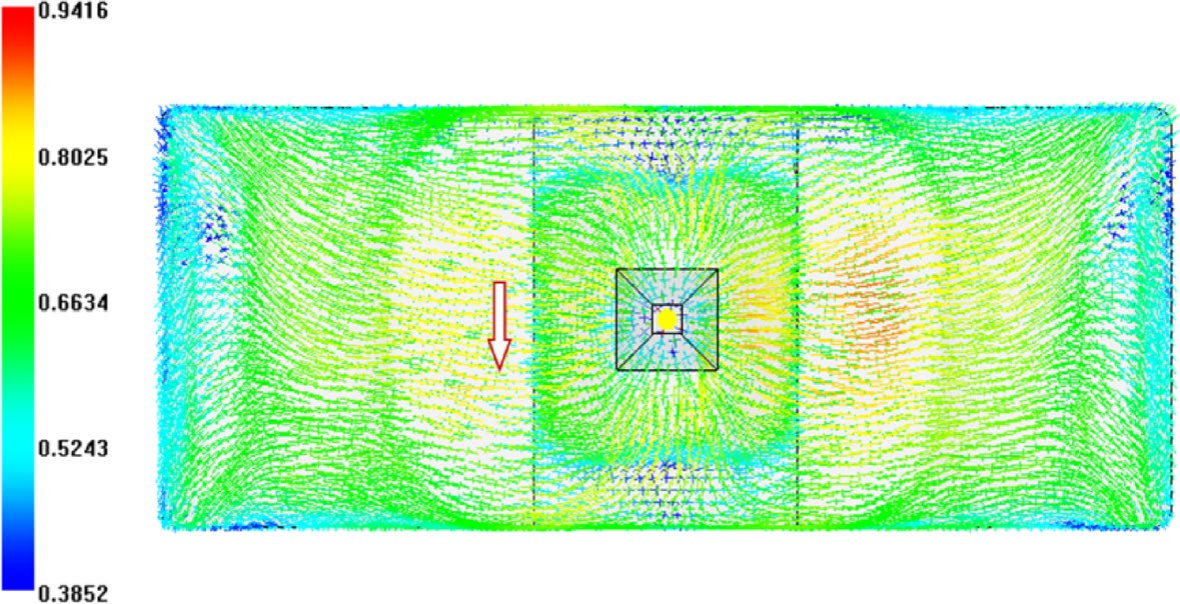
Fiber orientation distributions for horizontal injection molding.

The leaf spring clamp plate is a typical thick-walled structure, and gravity affects the melt flow during the filling phase in injection molding, subsequently affecting the fiber orientation distribution. To mitigate the impact of gravity on the filling pattern, the mold installation direction is altered from vertical to horizontal, and the samples are subsequently injection-molded in a horizontal plane, as shown in Figure 22. The process analysis predicts the fiber orientation distribution as shown in Figure 23, and the arrow indicates the direction of gravity. In comparison with Figure 10, the variability in fiber orientation diminishes, as evidenced by the diminishing red area, indicating a reduced influence of gravity on fiber orientation.

**Fig. 24:**
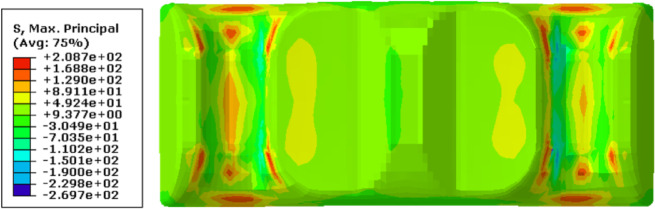
Stress field by FEA in the horizontally injection-molded leaf spring clamp plate.

Following the flowchart of the FEA in Figure 3, the maximum principal stress in the horizontally injection-molded leaf spring clamp plate is calculated to be 208 MPa, which is 23 MPa lower than the stress observed when the plate is molded vertically, as seen in Figure 24, the stress states in both end parts of the product are nearly identical. However, this maximum principal stress exceeds the allowable limit, indicating that the product fails to satisfy the strength criteria.

### Optimizing the plate wall thickness

**Fig. 25:**
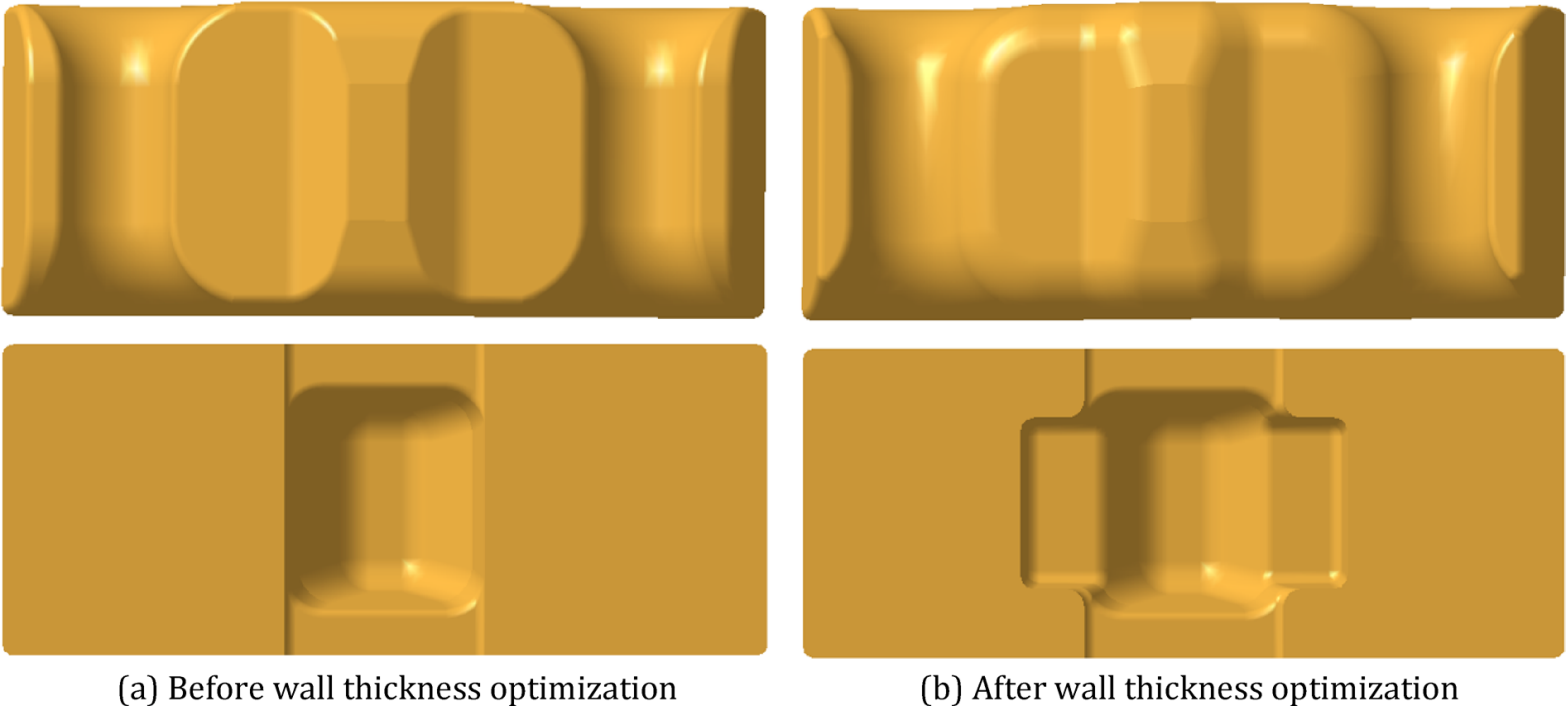
Structure layout of the injection-molded leaf spring clamp plate.

**Fig. 26:**
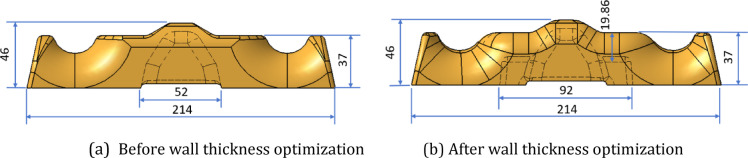
Profile of the injection-molded leaf spring clamp plate with the local thickness indicated.

The fiber orientation in injection-molded parts is affected by the complexity of the product structure in addition to gravity. The leaf spring clamp plate in this study has a thick and uneven wall thickness. Under the condition of maintaining the same appearance of the product, the wall thickness of the product is thinned, and grooves are dug to reduce the effect of the difference in wall thickness on the fiber orientation. The local thickness of the product was reduced from 37 mm to 19.86 mm, and the weight of the product was consequently reduced from 780 g to 680 g. The structural layouts and profiles of the product before and after wall thickness optimization are shown in Figure. 25 and Figure 26.

**Fig. 27:**
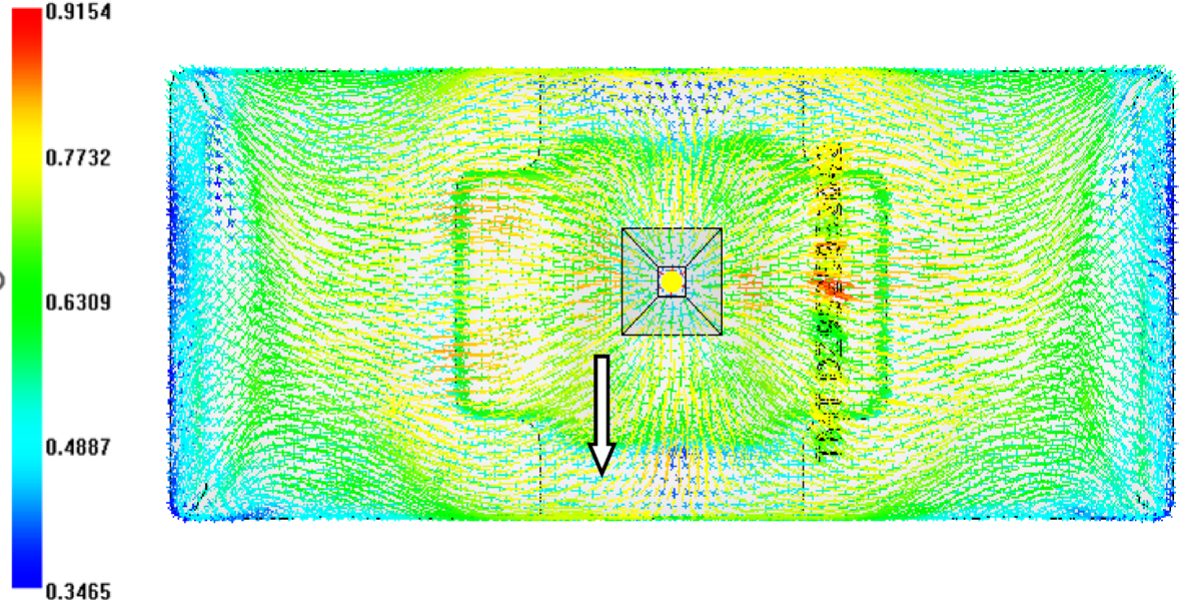
Fiber orientation distribution in the optimized product.

The horizontal injection molding process of the optimized plate is simulated. Figure 27 shows the fiber orientation tensor obtained from the simulation, indicating a uniform fiber orientation and no significant difference in fiber orientation between the fracture area of the original vertically injection-molded product and its symmetric site. The arrow indicates the direction of gravity.

**Fig. 28:**
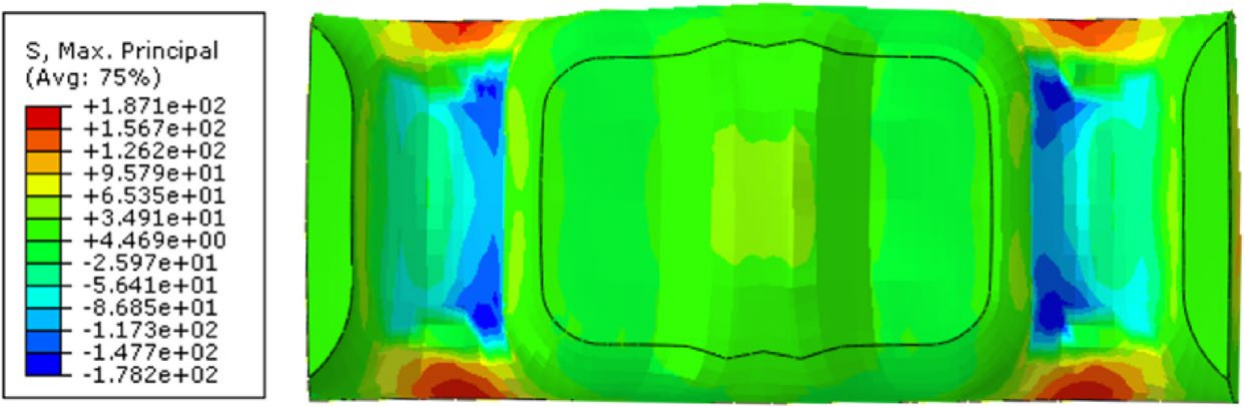
Finite element analysis results of the modified products.

Figure 28 shows the maximum principal stress field of the optimized product via FEA on the basis of the fiber orientation data in Figure 27. The analysis indicates that the maximum principal stress for the horizontal injection-molded product with the optimal wall thickness is 187 MPa, which is much lower than the material’s allowable stress of 205 MPa, and the stress states of the two end parts of the product are practically similar. Thus, the optimized product satisfies the strength criteria, moreover, its weight is concurrently lower than that of the original structure. Therefore, a modified product with an optimal wall thickness via horizontal injection molding is recommended.

### Verification testing

The samples used for verification tests were prepared following the prescribed recommended protocol and then positioned to replicate the actual automotive installation conditions, as shown in Figure 21(a). In the experiment, the preloading force was applied through the two U-bolts attached to the clamp plate, each with two preload screws. The preload force was applied alternately to each screw in incremental steps using a torque wrench rated at 1000 N·m, as outlined in Sect. 5.4, until a peak value of 260.25 kN was attained, culminating in a total preload force of 1041 kN and consequently a maximum tightening torque of 750 N·m, which is greater than the required value 700 N·m, thus ensuring the integrity of the leaf spring clamp plate assembly.

## Conclusions

This study presents a ​multiscale methodology​ for analyzing and optimizing the mechanical performance of ​composite clamp plates​ used in automotive leaf spring assemblies, with a focus on the ​fiber orientation distribution​ induced by injection molding. By integrating ​injection molding simulations, mesoscopic constitutive modeling, and macroscopic structural analysis, the failure mechanisms and optimization strategies of the leaf spring clamp plates are systematically investigated. The main research work and conclusions are summarized below:

(1) Root failure causes identification: For thick-walled products, the gravitational segregation during vertical injection molding leads to non-uniform fiber orientation, causing stress concentration in the injection molded products and resulting in premature fracture under loading.

(2) Design optimization and performance improvement: By switching the mounting direction of the injection mold from vertical to ​horizontal and reducing the local wall thickness, the ​maximum principal stress in the molded clamp plate decreases by 19% (from 231 MPa to 187 MPa)​, whereas the ​weight is reduced by 12.8% (from 780 g to 680 g)​, achieving ​simultaneous strength enhancement and lightweighting.

While this study provides a ​robust framework​ for optimizing fiber-reinforced composite components, future work should address anisotropy in material modeling, process complexities, dynamic loading, and industrial scalability​ to fully realize its potential in automotive and aerospace applications. For material modeling, the current approach assumes tensile‒compressive symmetry and maximum principal stress failure criteria, which neglects the ​differences in tensile and compressive strengths​ and the anisotropic failure modes (e.g., matrix cracking under tension vs. delamination under compression) often observed in fiber-reinforced composites, modeling of compressive mechanical properties and anisotropy is essential to account for the potential asymmetry in future research. Advanced failure criteria (e.g., ​Hashin’s criteria, ​Tsai-Wu, or ​Puck’s criteria) that account for ​fiber-dominated and matrix-dominated failure modes​ under combined loading are suggested in future work for accurate failure prediction​ in real-world applications, where components experience multiaxial loads and variable stress states. Furthermore, only ​static loading​ cases are considered in this study, and it is necessary to extend the FEA to include ​cyclic loading (e.g., road vibrations) and ​impact scenarios​ to assess the long-term durability of the leaf spring clamp plate assembly under real-world conditions.

## Data Availability

Data sets generated during the current study are available from the first author (Feng Wang, wangfeng4@csrzic.com) on reasonable request.
